# CARD9 protein SUMOylation regulates HOXB5 nuclear translocation and Parkin‐mediated mitophagy in myocardial I/R injury

**DOI:** 10.1111/jcmm.70195

**Published:** 2024-11-04

**Authors:** Yuanbin Li, Yuting Tang, Xu Yan, Hui Lin, Wanjin Jiang, Luwei Zhang, Hu Zhao, Zhuang Chen

**Affiliations:** ^1^ Department of Medicine Hunan Traditional Chinese Medical College Zhuzhou Hunan People's Republic of China; ^2^ Department of Pathology The Affiliated Cancer Hospital of Xiangya School of Medicine, Central South University/Hunan Cancer Hospital Changsha Hunan People's Republic of China; ^3^ Department of Cardiovascular The Affiliated Hospital of Hunan Academy of Traditional Chinese Medicine Changsha Hunan People's Republic of China

**Keywords:** CARD9, HOXB5 nuclear, Mitophagy, O‐GlcNAc glycosylation, SUMOylation, translocation

## Abstract

Myocardial injury induced by ischemia–reperfusion (I/R) remains a difficult clinical problem. However, the exact mechanisms underlying I/R‐induced have yet to be clarified. CARD9 is an important cytoplasmic‐binding protein. In this study, an immunocoprecipitation assay showed that SUMOylation of the CARD9 protein promoted the binding of CARD9 to HOXB5, but hindered the O‐GlcNAc glycosylation of HOXB5, a predicted transcription factor of Parkin and a key factor in mitophagy. O‐GlcNAc glycosylation is an important signal for translocation of proteins from the cytoplasm to the nucleus. CARD9 protein SUMOylation is regulated by PIAS3, which is related to I/R‐induced myocardial injury. Therefore, we propose that knockdown of PIAS3 inhibits SUMOylation of the CARD9 protein, facilitates the dissociation of CARD9 and HOXB5, which increases the O‐GlcNAc‐mediated glycosylation of HOXB5, while the resulting HOXB5 nuclear translocation promotes Parkin‐induced mitophagy and alleviates myocardial I/R injury.

## 1 INTRODUCTION

The pathogenesis of myocardial ischemia/reperfusion (I/R) injury remains only incompletely understood. Currently, I/R injury is believed to be predominantly related to mitochondrial dysfunction, oxidative stress, and autophagy.^1^ Furthermore, accumulating evidence suggests that autophagy (also known as macroautophagy) plays an important role in maintaining cardiac function following I/R. Mitochondrial autophagy (mitophagy) is a particular type of autophagy regulated by signals such as PINK1‐Parkin. In this process, damaged mitochondria are ‘tagged’ and surrounded by autophagosomes, which are ultimately disassembled in lysosomes. Prior studies have shown that mitophagy activation significantly inhibits I/R‐induced cardiomyocyte death, reduces the size of myocardial infarction, and alleviates post‐I/R myocardial dysfunction, indicating that mitophagy exerts a cardioprotective effect.^2^, ^3^

Our previous study reported that dysfunctional autophagy in cardiomyocytes hinders autophagic flux and promotes I/R‐induced myocardial injury, and that CARD9 protein alleviates I/R‐induced myocardial injury through the regulation of autophagy.^4^ Although CARD9 is known to induce autophagy in cardiomyocytes, its regulatory effect on mitophagy remain unclear. As an important intracellular bridging protein, the biological functions of CARD9 are inextricably linked to those of its bound proteins. HOXB5 is a homeobox protein involved in the regulation of lung, heart, nerve and vascular development.^5^, ^6^, ^7^ Bioinformatic analysis has previously suggested that HOXB5 has a strong binding capacity to the Parkin gene promoter (data not shown). However, the molecular mechanisms regulating the binding between CARD9 and HOXB5 require further investigation.

Protein function depends on the regulation of post‐translational protein modifications. For example, protein kinase activity is predominantly regulated by protein phosphorylation modifications, while protein–protein binding is influenced by protein SUMO modifications (also known as SUMOylation), and protein shuttling between the cytoplasm and nucleus is regulated by O‐GlcNAc glycosylation modifications.^8^, ^9^ I/R further induces protein O‐GlcNAc glycosylation modification.^10^, ^11^ SUMOylation was originally thought to be a class of ubiquitination, but later studies have shown that in most cases, SUMO does not affect protein degradation, instead influencing protein–protein binding.^8^, ^12^ The expression of PIAS3, a SUMO E3 ligase, is elevated in many cancerous tissues, including cervical, prostate, osteosarcoma, lung, breast, and brain cancers.^13^ In addition, bioinformatic analysis has suggested that HOXB5 contains multiple O‐GlcNAc glycosylation modification sites.

In summary, based on our previous research base and literature review, we propose the following hypotheses: (1) SUMOylation of CARD9 promotes the binding of CARD9 to HOXB5, subsequently inhibiting the O‐GlcNAc glycosylation of HOXB5; (2) O‐GlcNAc glycosylation modification promotes the translocation of HOXB5 to the nucleus, which can promote mitophagy following I/R by enhancing the expression of Parkin; (3) Knockdown of PIAS3 downregulates the levels of SUMOylated CARD9, promotes HOXB5‐induced mitophagy in cardiomyocytes, and alleviates I/R‐induced myocardial injury.

## 2 MATERIALS AND METHODS

### 2.1 Vector construction, packaging and transfection

Knockdown and overexpression vectors for PIAS3 and HOXB5, as well as knockdown vectors for CARD9, OGT and Parkin were constructed by Genechem Co., Ltd. (Shanghai, China). The vectors were transfected into cardiomyocytes in vitro using Lipofectamine 3000 (Invitrogen). H9c2 cells were further transfected with control small interfering RNA (siRNA) or Parkin, OGT siRNA duplex (RiboBio) and riboFECTTM CP (RiboBio). In vivo, knockdown vectors for PIAS3 and HOXB5 were packaged with adenovirus (Genechem, China), diluted with Invivofectamine 3.0 reagent and injected into the tail veins of rats.

### 2.2 Construction of myocardial I/R model

The method for the construction of myocardial I/R model referred to previous study.^4^ Sprague Dawley (SD) rats were anaesthetised with 2% isoflurane ether and then placed in supine position on a heating pad maintained at 37°C. After connecting to the ventilator, left thoracotomy was performed, in which the subcutaneous tissues, pectoralis major, and serratus anterior were bluntly detached, and the heart was rapidly extruded to expose the left anterior descending branch of the coronary artery (LAD), which was ligated with a 6–0 wire for 30 min to induce ischemia. The wire was subsequently tied into a live knot and left outside the thoracic cavity by 3–4 cm. Following ligation, the ventricles below the ligated area turned pallor or cyanotic, and the ligated wire was slowly pulled out after 30 min to restore the myocardial blood supply for 24 h. No LAD ligation was performed in the sham group. All mice were maintained in a specific pathogen‐free animal facility, and handled in accordance with the relevant guidelines (No. 20230301) for the Care and Use of Laboratory Animals.

### 2.3 Assessment of cardiac function by echocardiography

Cardiac function was evaluated using a transthoracic two‐dimensional echocardiogram (Vinno Corporation, VINNO 6 LAB, Suzhou, CHINA), equipped with a 23‐MHz mechanical transducer. VINNO Analysis software was used to assess the left ventricular internal dimension diastole (LVIDd), left ventricular internal dimension systole (LVIDs), percent LV fractional shortening (LVFS), and LV ejection fraction (LVEF).

### 2.4 Assessment of infarct size by Evans blue and TTC staining

At the end of reperfusion, rats were anaesthetised again, and the ventilator was connected. The LAD was then ligated again, 2% Evans blue (Sigma E2129) was rapidly injected into the femoral vein, the lip and extremity skin of the rats were stained blue, and then the residual dye in the ventricular cavity was removed with filter paper, after which the hearts were rapidly removed and frozen at −40°C. The frozen hearts were then cut into five circular slices averaging 2 mm in thickness, from the base to the apex. The slices of the left heart were then placed in 1% TTC (dissolved in PBS, Sigma, T8877) at 37°C in the dark for 15 min, and then fixed in 10% methanol solution for 24 h. Finally, the non‐ischemic myocardial tissue was stained blue, the non‐infarcted myocardial tissue at risk was stained red, and the infarcted myocardial tissue was left unstained and white. After scanning the heart sections, the blue, red and white areas were determined using Image‐Pro Plus 6.0 analysis software. The left ventricular (LV) area is the sum of the blue, red, and white areas. The total area at risk (AAR) was defined as the sum of the red and white areas. The infarct area was white. The following calculations were applied: Percentage area‐at‐risk = AAR/LV × 100%. Infarct area percentage = infarct area/AAR × 100%.

### 2.5 Construction of the cardiomyocyte H/R model and infection with adenovirus

Cardiomyocytes were inoculated into gelatin‐coated 6‐well plates at a density of 1 × 10^6^ cells/well and cultured in DMEM containing 10% fetal bovine serum for 36 h. H9c2 cells were exposed to adenoviruses AdVector, AdshPIAS3 or AdshHOXB5 for 8 h. Following adenovirus transduction, the method for the construction of cardiomyocyte H/R model referred to previous study.^14^ The medium was replaced with fresh DMEM supplemented with 10% FBS, and hypoxia/reoxygenation (H/R) experiments were conducted 48 h later. The cells were then transferred to a hypoxic (<1% O_2_, 5% CO_2_) incubator (ESCO, Taicang, China) in serum‐ and glucose‐free fresh medium for 6 h, and then incubated in a normal (21% O_2_, 5% CO_2_) incubator for 2 h in DMEM containing 10% FBS.

### 2.6 Immunoprecipitation

Cells were lysed in lysis buffer supplemented with protease inhibitors for 30 min at 4°C, and centrifuged at 12000*g* for 10 min at 4°C, after which the supernatant collected and incubated overnight at 4°C with anti‐CARD9, anti‐HOXB5 antibodies and 50 μL protein A/G beads. Following the immunoprecipitation reaction, the bead‐antigen–antibody complex was collected by centrifugation at 3000*g* for 5 min at 4°C. The supernatant was removed, and the cells were washed three times with lysis buffer. Finally, 60 μL of 2× SDS buffer was added and boiled for 10 min to denature the proteins, which were separated by SDS‐PAGE and subsequently subjected to Western blot to detect the expressions of SUMO1, SUMO2/3, O‐GlcNAc and HOXB5.

### 2.7 Chromatin immunoprecipitation (ChIP)

Binding of the Parkin gene promoter was assayed. Cells were lysed in lysis buffer A and sonicated to generate 200–500 bp DNA fragments. The ‘protein‐DNA’ complex was precipitated with HOXB5 antibody, and DNA products were eluted and assessed by qPCR for Parkin promoter binding. Intensity data are presented as the percentages of the input.

### 2.8 Dual luciferase reporter gene assay

To detect the transcriptional activation of Parkin by HOXB5, cardiomyocytes were co‐transfected with each plasmid. The cell lysate containing the reporter gene was then thoroughly mixed and lysed, and centrifuged at 10,000–15,000 *g* for 3–5 min, after which the supernatant was extracted for the assay. Subsequently, 100 μL of luciferase assay reagent was added, after which the relative light units (RLU) were assessed following gunshot homogenization. After completing the firefly luciferase assay, we added an equal amount of sea kidney luciferase assay working solution to determine the RLU, calculated the ratio of the two luciferase enzymes (reporter gene/internal reference gene), and compared the ratio differences between the different groups.

### 2.9 Fluorescent probe tracks mitophagy

The dynamics of mitochondrial autophagy were tracked using Mito Tracker, a mitochondria‐specific fluorescent probe and Lyso Tracker, a lysosome‐specific fluorescent probe. Before the experiment, the appropriate concentrations of Mito‐Tracker and Lyso‐Tracker probe working solutions were prepared, the cell culture medium was removed, and the cells were incubated with the two kinds of probe working solutions at 37°C for 5–30 min. The fluorescence intensity and co‐localization of mitochondrial and lysosomal fluorescence were further observed using a fluorescence microscope to reflect the dynamic changes in mitophagy in cells.

### 2.10 Electron microscopic observation of mitophagy

Transmission electron microscopy was performed to observe mitochondrial autophagy. The cells were fixed with 2.5% glutaraldehyde for 2 h, followed by 2% osmic acid for 2 h. The cells were then dehydrated in graded ethanol (50%, 70%, 80%, 90% and 100%), embedded in acrylic resin, sectioned in ultrathin (50–70 nm) sections on a microtome, double‐stained with 3% uranyl acetate‐lead citrate, and observed and photographed using electron microscopy at 80 KV.

### 2.11 Western blot analysis

Heart tissues or cardiomyocytes were collected and added to a lysis buffer containing PMSF (Dingguo Biotechnology, Beijing, China). The tissue was then mechanically homogenized, mixed with oscillation, placed on ice for 30 min, and centrifuged at 12000 rpm at 4°C for 30 min. The supernatant was then collected and the protein concentration was determined using the BCA method. Protein samples were denatured with 2× loading buffer at 100°C for 10 min, after which samples were loaded at 30 μg/well. Following SDS‐PAGE, proteins were transferred onto a PVDF membrane (Millipore, Bedford, MA, USA), which was subsequently blocked in 5% skim milk powder for 2 h at room temperature before being incubated overnight at 4°C with anti‐GAPDH, SUMO1, LC3 (1:3000; Sigma‐Aldrich), O‐GlcNAc, CARD9, PINK1, Parkin, PIAS3, HOXB5, pSer65‐Parkin and pSer65‐Ub antibodies (1:3000; CST, USA). The next day, after washing with TBST, the appropriate horseradish peroxidase‐labelled secondary antibody (BOSTER, Wuhan, China) was added and incubated for 1 h at room temperature. ECL (Thermo Fisher Scientific, Waltham, MA, USA) exposure was performed after washing in TBST. An Alpha Imager 2200 was used to calculate the optical density of each strip and to analyse the expression of multiple proteins.

### 2.12 Flow cytometric (FCM) analysis and TUNEL staining for apoptosis detection

FCM analysis was performed to evaluate the levels of cellular apoptosis by staining H9c2 cells with propidium iodide (PI) and annexin V‐fluorescein isothiocyanate (FITC) (Roche Ltd., Basel, Switzerland), according to the manufacturer's instructions. H9c2 cells were transfected with plasmids, subjected to H/R, and stained with Annexin V‐FITC/PI for 5 min. The cell samples were analysed using FCM and FlowJo software.

TUNEL staining was assessed using the In Situ Cell Death Detection Kit, POD (Roche Ltd., Basel, Switzerland). Briefly, rat heart sections were stained according to the manufacturer's instructions. Six fields in each section were randomly selected to count apoptotic cells using a fluorescence microscope (EVOS FL Auto; Thermo Fisher Scientific, USA). The number of apoptotic cells was then calculated.

### 2.13 Statistical analysis

Data are expressed as the mean ± standard deviation (SD). Data were analysed using an unpaired Student's *t*‐test for two independent groups and one‐way ANOVA for multiple groups using SPSS 22.0. Bonferroni's post hoc test was applied after ANOVA for multiple comparisons (for three or more groups) to minimize type I errors, as appropriate. Differences were considered statistically significant at a two‐tailed *p* < 0.05.

## 3 RESULTS

### 3.1 PIAS3 knockdown regulates the SUMOylation of CARD9 and O‐GlcNAc glycosylation of HOXB5

**FIGURE 1:**
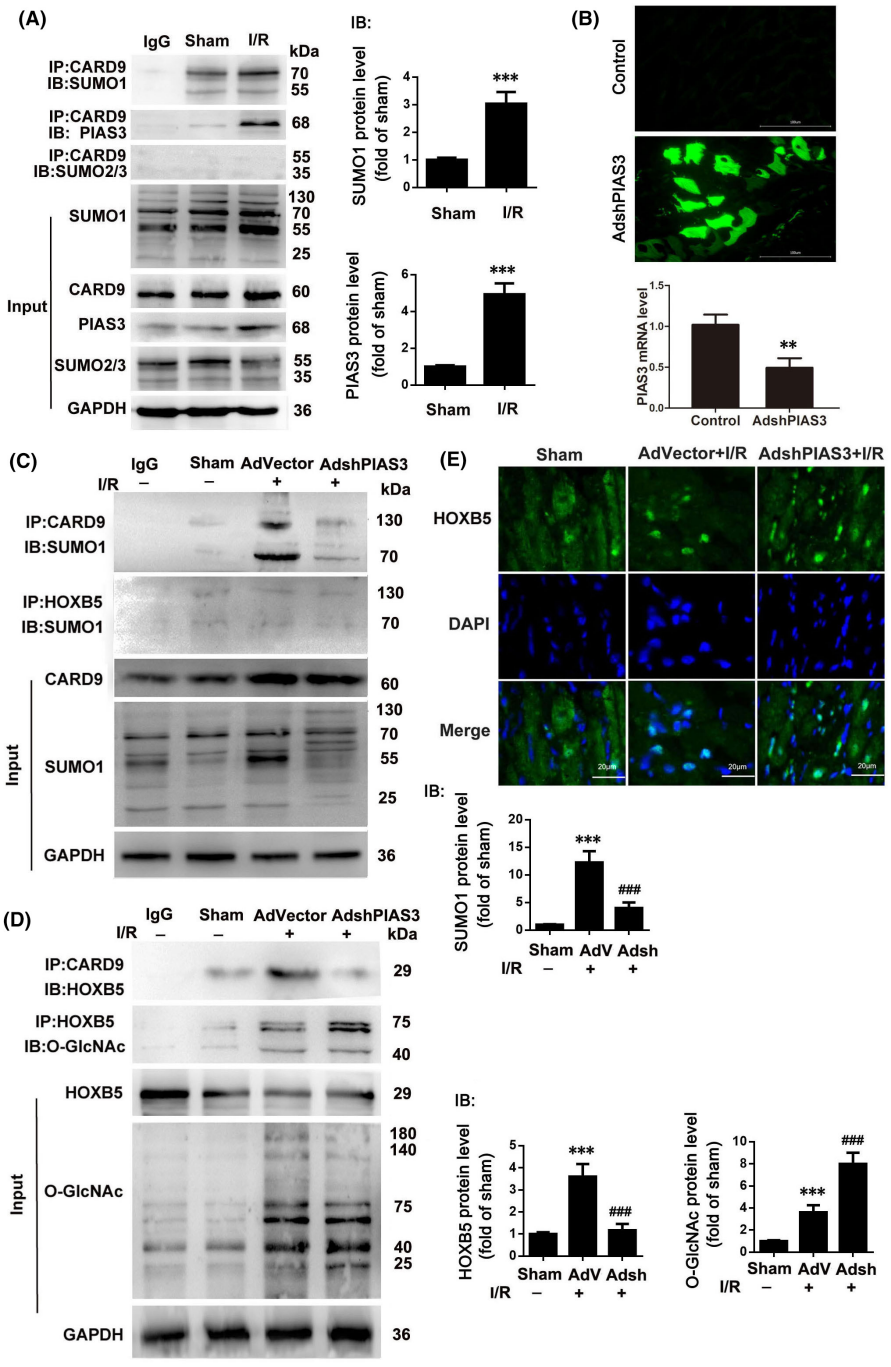
Knockdown of PIAS3 regulates SUMOylation and O‐GlcNAc glycosylation of the CARD9 and HOXB5 proteins. (A) Immunoprecipitation of dissociated heart tissue after I/R with anti‐CARD9 antibodies was performed, and subjected to Western blotting with SUMO1, SUMO2/3 and PIAS3 antibodies. (B–E) Rats were infected with empty (AdVector) or PIAS3 knockdown vectors (AdshPIAS3) (B) with or without I/R, and the resulting lysates were immunoprecipitated with anti‐CARD9 antibody or anti‐HOXB5 antibody. The SUMOylation of CARD9 or HOXB5 was analysed by Western blotting (C). The CARD9 or HOXB5 immunocomplex was then probed for HOXB5 and O‐GlcNAc proteins (D). IB, The binding proteins. Representative fluorescent images showing the translocation of HOXB5 from the cytoplasm to the nucleus in cardiac tissue (E). Scale bar, 20 μm. *n* = 6. **p < 0.01 versus Control, ***p < 0.001 versus Sham, ^###^ *p* < 0.001 versus AdVector+I/R.

We first constructed an I/R rat model, which showed that I/R induced an increase in intracellular SUMO1 and PIAS3 protein levels (input), as well as an increase in PIAS3 protein levels (IB) and SUMO1 levels (IB) on the CARD9 protein (Figure 1A). H9c2 cardiomyocytes were then transfected with a knockdown‐negative vector (AdVector) or PIAS3 knockdown vector (AdshPIAS3) (Figure 1B). Immunoprecipitation revealed that I/R treatment increased the level of SUMO1 attached to the CARD9 protein, but had no significant effect on the level of SUMO1 attached to the HOXB5 protein; meanwhile, knockdown of PIAS3 downregulated the levels of SUMO1 in the CARD9 protein (Figure 1C). I/R treatment promoted CARD9 protein binding to HOXB5, and resulted in a detectable level of HOXB5 protein O‐GlcNAc glycosylation. PIAS3 knockdown inhibited CARD9 protein binding to HOXB5 protein, but dramatically increased HOXB5 protein O‐GlcNAc glycosylation (Figure 1D). Immunofluorescence revealed that PIAS3 knockdown promoted the translocation of HOXB5 from the cytoplasm to the nucleus after I/R (Figure 1E).

### 3.2 CARD9 SUMOylation facilitates the binding of CARD9 to HOXB5 and inhibits the O‐GlcNAc glycosylation of HOXB5

**FIGURE 2:**
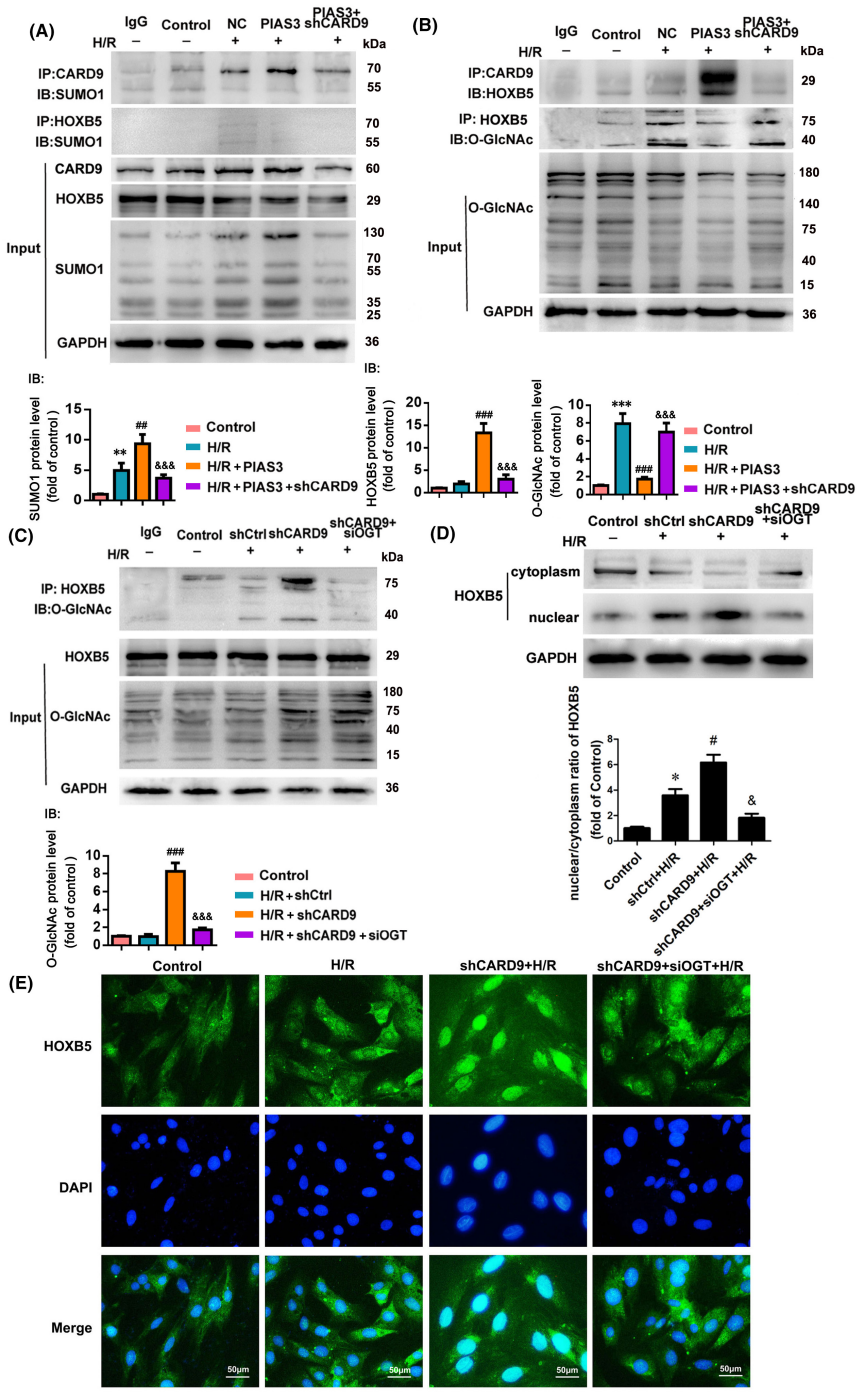
CARD9 SUMOylation facilitates the binding of CARD9 to HOXB5, inhibiting O‐GlcNAc glycosylation and nuclear translocation of HOXB5. (A, B) H9c2 cells transfected with normal control vectors (NC) or PIAS3 overexpression vectors (PIAS3). H9c2 cells were co‐transfected with PIAS3 and shRNA‐CARD9 (shCARD9) for 48 h prior to H/R exposure. Cell lysates were subsequently immunoprecipitated with anti‐CARD9 and anti‐HOXB5 antibodies. Endogenous SUMOylation of CARD9 or HOXB5 was analysed by Western blotting (A). Endogenous HOXB5 and O‐GlcNAc glycosylation of HOXB5 was analysed by Western blotting (B). IB, The binding proteins. (C–E) H9c2 cells transfected with shRNA‐control (shCtrl) or shRNA‐CARD9 (shCARD9). H9c2 cells were co‐transfected with shCARD9 and siRNA‐OGT (siOGT) for 48 h prior to H/R exposure. Cell lysates were subsequently immunoprecipitated with anti‐HOXB5 antibodies. Endogenous O‐GlcNAc glycosylation of HOXB5 was analysed by Western blotting (C). The levels of HOXB5 in the cytoplasm and nucleus were detected by Western blotting (D). IB, The binding proteins. *n* = 6. *p < 0.05, **p < 0.01 and ***p < 0.001 versus Control, ^#^ *p* < 0.05 and ^###^ *p* < 0.001 versus NC + H/R or shCtrl+H/R, ^&^ *p* < 0.05 and ^&&&^ *p* < 0.001 versus PIAS3 + H/R or shCARD9 + H/R. The representative fluorescent images for the translocation of HOXB5 from the cytoplasm to the nucleus in H9c2 cells (E). Scale bar, 20 μm. NC, empty vector. shCtrl, shRNA‐control.

Previously, we showed that the knockdown of PIAS3 downregulated the SUMO level of the CARD9 protein in cardiomyocytes following I/R in vivo, and further reduced the level of HOXB5 protein co‐immunoprecipitated with the CARD9 protein, which was increased by O‐GlcNAc glycosylation. To further elucidate the role of SUMO in the regulation of CARD9 binding to HOXB5 and the effect of CARD9 binding to HOXB5 on O‐GlcNAc glycosylation, PIAS3‐overexpression (PIAS3) was performed in H9c2. As expected, overexpression of PIAS3 increased the levels of CARD9 SUMOylation and interaction of HOXB5 with CARD9, while downregulating the O‐GlcNAc glycosylation level of HOXB5 (Figure 2A,B). In addition, following PIAS3 overexpression and CARD9 knockdown in H9c2 cells, the O‐GlcNAc glycosylation level of HOXB5 increased (Figure 2B).

To confirm this hypothesis, we knocked down CARD9 alone or in combination with O‐GlcNAc glycosyltransferase (OGT) in H9c2 cells and subsequently subjected them to H/R treatment. Knockdown of CARD9 liberated the O‐GlcNAc glycosylation site of HOXB5, and promoted the translocation of HOXB5 to the nucleus. However, simultaneous knockdown of CARD9 and OGT blocked the O‐GlcNAc glycosylation of HOXB5 and inhibited its translocation to the nucleus. Moreover, HOXB5 protein was found to be predominantly located in the nucleus in CARD9‐deficient cells, although some HOXB5 was also identified in the cytoplasm in both CARD9 and OGT knockdown cells (Figure 2C–E). These results indicate that CARD9 knockdown increased the O‐GlcNAc glycosylation and nuclear translocation of HOXB5.

### 3.3 O‐GlcNAc glycosylation of HOXB5 enhances Parkin expression to promote mitophagy in cardiomyocytes after H/R

**FIGURE 3:**
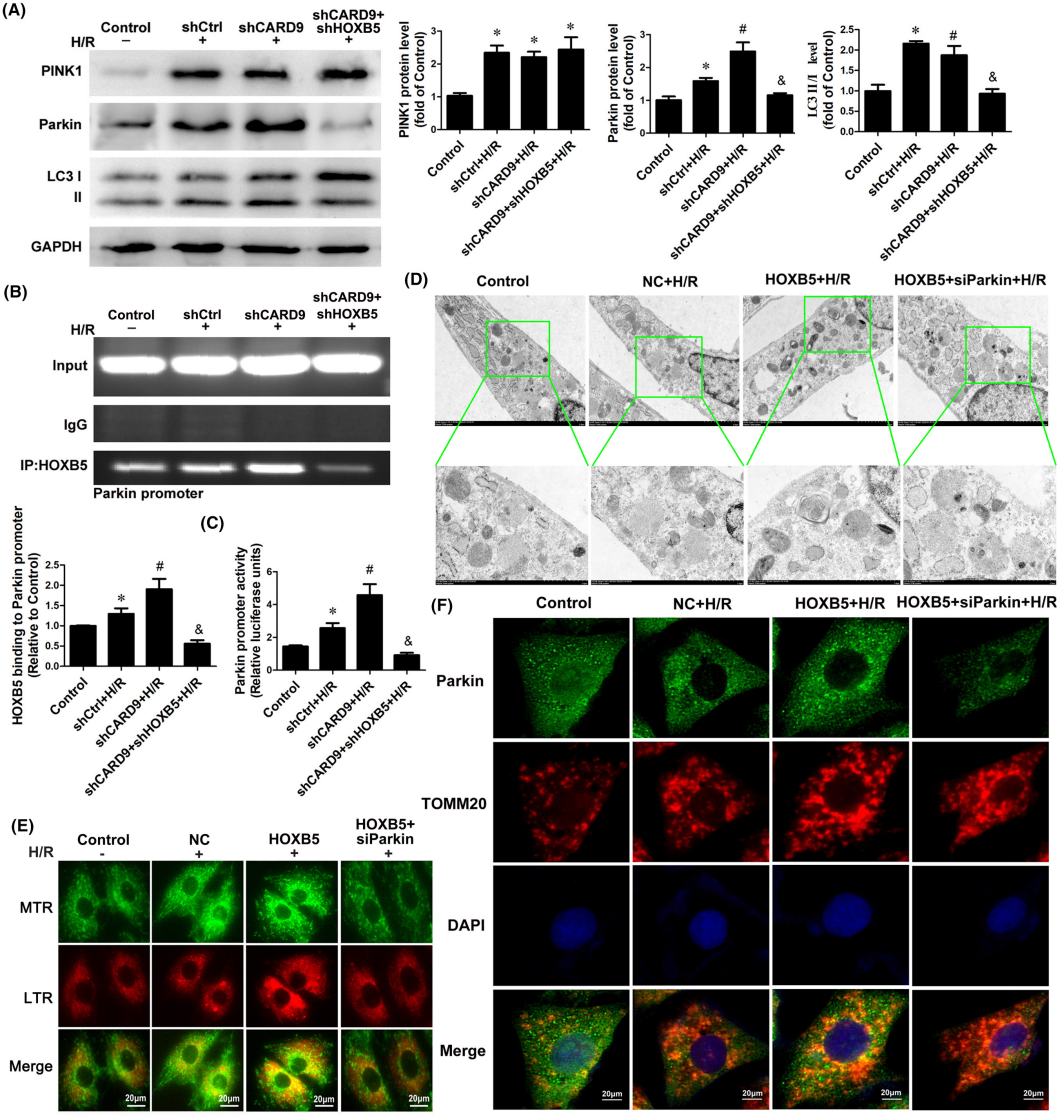
O‐GlcNAc glycosylation of HOXB5 enhances Parkin expression to promote mitophagy in cardiomyocytes following H/R. (A–C) H9c2 cells were transfected with shCARD9 with or without shRNA‐HOXB5 (shHOXB5) for 48 h prior to H/R exposure. The expression levels of Pink1 and Parkin, as well as the LC3‐II/I ratio, were examined by Western blotting (A). *n* = 6. *p < 0.05 versus Control, ^#^ *p* < 0.05 versus shCtrl+H/R, ^&^ *p* < 0.05 versus shCARD9 + H/R. Vinding of HOXB5 to the Parkin promoter in H9c2 cells using chromatin immunoprecipitation (B). *n* = 6. *p < 0.05 versus Control, ^#^ *p* < 0.05 versus shCtrl+H/R, ^&^ *p* < 0.05 versus shCARD9 + H/R. H9c2 cells were transfected with the Parkin promoter reporter gene (−750 to +145 bp), and luciferase assays were performed. The reporter activities are shown as the relative luciferase units (RLU) normalized to the pRL‐TK vector activity (C). *n* = 6. *p < 0.05 vs. Control, ^#^ *p* < 0.05 vs. shCtrl+H/R, ^&^ *p* < 0.05 versus shCARD9 + H/R. (D–F) H9c2 cells were co‐transfected with HOXB5 overexpression vector (HOXB5) and siRNA‐Parkin (siParkin) for 48 h prior to H/R exposure. Electronic micrographs of autophagic vacuoles containing mitochondria (entire or almost degraded) in H9c2 cells (D). Scale bar, 1 μm. Representative images of Mito Tracker (MTR, green fluorescent) and Lyso Tracker (LTR, red fluorescent) stained puncta in H9c2 cells (E). Scale bar, 20 μm. The co‐localization efficiency between Parkin and TOMM20 was quantified and analysed (right panel) (F). Scale bar, 20 μm.

To clarify the mechanism by which HOXB5 nuclear translocation can promote mitophagy in cardiomyocytes following H/R, we knocked down CARD9, overexpressed HOXB5, knocked down both CARD9 and HOXB5, or overexpressed HOXB5 together with parkin knockdown in H9c2 cells. These modified cell lines were subsequently subjected to H/R treatment. Western blotting revealed that H/R upregulated PINK1 and Parkin protein levels and the LC3II/I ratio, while CARD9 knockdown further increased Parkin protein levels, but had no significant effect on PINK1 levels; however, both CARD9 and HOXB5 knockdown decreased the expression of Parkin (Figure 3A). Moreover, our investigation confirmed that CARD9 knockdown increased HOXB5 binding to the parkin promoter compared to H/R alone, whereas HOXB5 deficiency reduced the interaction of HOXB5 with the Parkin promoter (Figure 3B). To investigate the effect of HOXB5 on Parkin promoter activity, a luciferase reporter gene assay was performed. Parkin promoter activity was found to be significantly increased in CARD9 knockdown H9c2 cells following H/R treatment compared to that in the empty vector cells. However, the reporter gene expression was significantly reduced in both CARD9 and HOXB5 knockdown cells (Figure 3C). To determine the role of HOXB5 overexpression in mitophagy and the potential interdependence between HOXB5 and Parkin, we used transmission electron microscopy to observe mitophagy, with the result showing that HOXB5 overexpression increased the formation of autophagic vacuoles containing mitochondria (entire or almost degraded) following H/R injury. Further, aberrant mitochondrial morphology, mitochondrial swelling, and disordered arrangement were observed in Parkin‐deficient cells modified to overexpress HOXB5 (Figure 3D). Meanwhile, H/R treatment slightly increased the intensity of yellow light in the cells, while the overexpression of HOXB5 further increased the intensity of yellow light in the cells after H/R. However, the silencing of parkin reversed the effect of HOXB5 overexpression (Figure 3E). To clarify whether Parkin binds to mitochondria, we used antibodies to label Parkin and the mitochondria‐specific protein TOMM20. Immunofluorescence further revealed that the overexpression of HOXB5 further promoted Parkin binding to mitochondria following H/R (Figure 3F). These data indicated that parkin is a direct target for the transcriptional regulation of HOXB5. HOXB5 overexpression further promotes parkin transcription and mitophagy after H/R.

### 3.4 Knockdown of HOXB5 suppresses mitophagy and exacerbated the cardiac dysfunction after I/R

**FIGURE 4:**
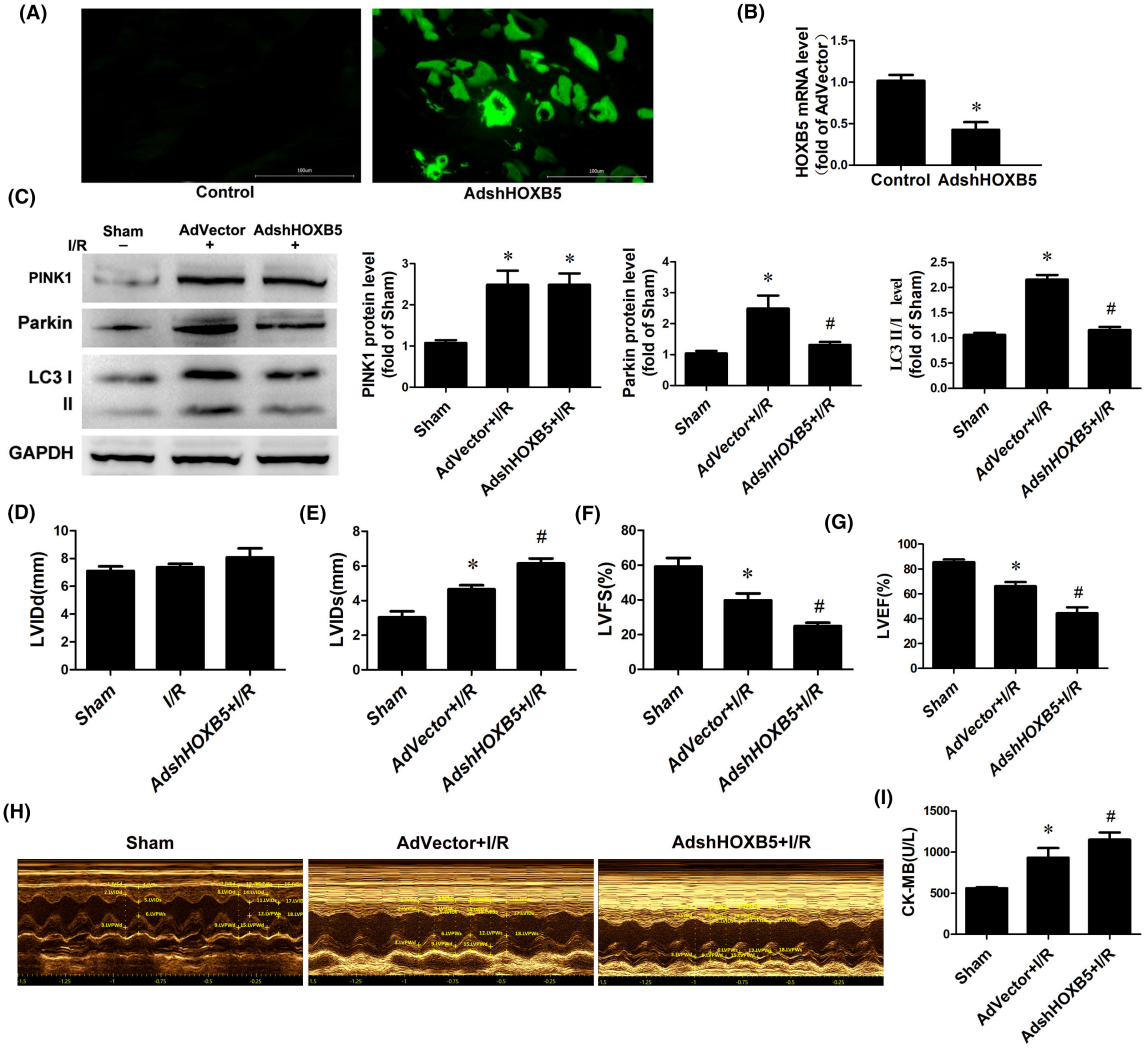
The knockdown of HOXB5 suppressed mitophagy and exacerbated cardiac dysfunction after M‐I/R. (A–I) Rat hearts infected with adenovirus‐mediated vector control (AdVector) or adenovirus‐mediated HOXB5 knockdown (AdshHOXB5), representative images of HOXB5 (green fluorescent) and q‐PCR analysis of HOXB5 mRNA (A, B). *n* = 6. *p < 0.05 versus Control. Immunoblot analysis of Pink1 and Parkin protein levels and the LC3‐II/I ratio in hearts of AdVector and AdshHOXB5 infected rats after M‐I/R (C). *n* = 6. *p < 0.05 versus Sham, ^#^ *p* < 0.05 versus AdVector+I/R. Echocardiographic measurement of left ventricular internal dimension diastole (LVIDd) (D), left ventricular internal dimension systole (LVIDs) (E), fractional shortening (F), and ejection fraction (G) in hearts infected with AdVector or AdshHOXB5. *n* = 6. *p < 0.05 versus sham, ^#^ *p* < 0.05 versus AdVector+I/R. Serum levels of cardiac creatine kinase (CK) at 24 h post‐reperfusion in AdVector and AdshHOXB5 rats (I). *n* = 6. *p < 0.05 versus sham, ^#^ *p* < 0.05 versus AdVector+I/R.

To determine whether HOXB5 influences heart function in rats subjected to I/R, HOXB5 was knocked down using adenoviral RNAi (AdshHOXB5) (Figure 4A,B). Western blotting showed that I/R upregulated PINK1 and Parkin protein levels and the LC3II/I ratio, whereas HOXB5 knockdown decreased Parkin protein levels and the LC3II/I ratio but had no significant effect on PINK1 levels (Figure 4C). We investigated whether HOXB5 knockdown affected cardiac function in rats after M‐I/R. Doppler echocardiography further revealed a significant reduction in cardiac function, as indicated by a relatively longer LVIDd and relatively lower LVFS and LVEF in the ventricular systolic period compared to I/R rats (Figure 4D–H). Furthermore, serum creatine kinase (CK) levels were found to be significantly elevated in HOXB5‐deficient rats subjected to I/R injury compared with those in I/R‐injured rats (Figure 4I). It is not difficult to see that HOXB5 knockdown exacerbated myocardial contractile dysfunction rather than diastolic function in rats following I/R.

### 3.5 Knockdown of PIAS3 enhances the PINK1/Parkin kinase activity and promotes HOXB5‐mediated mitophagy

**FIGURE 5:**
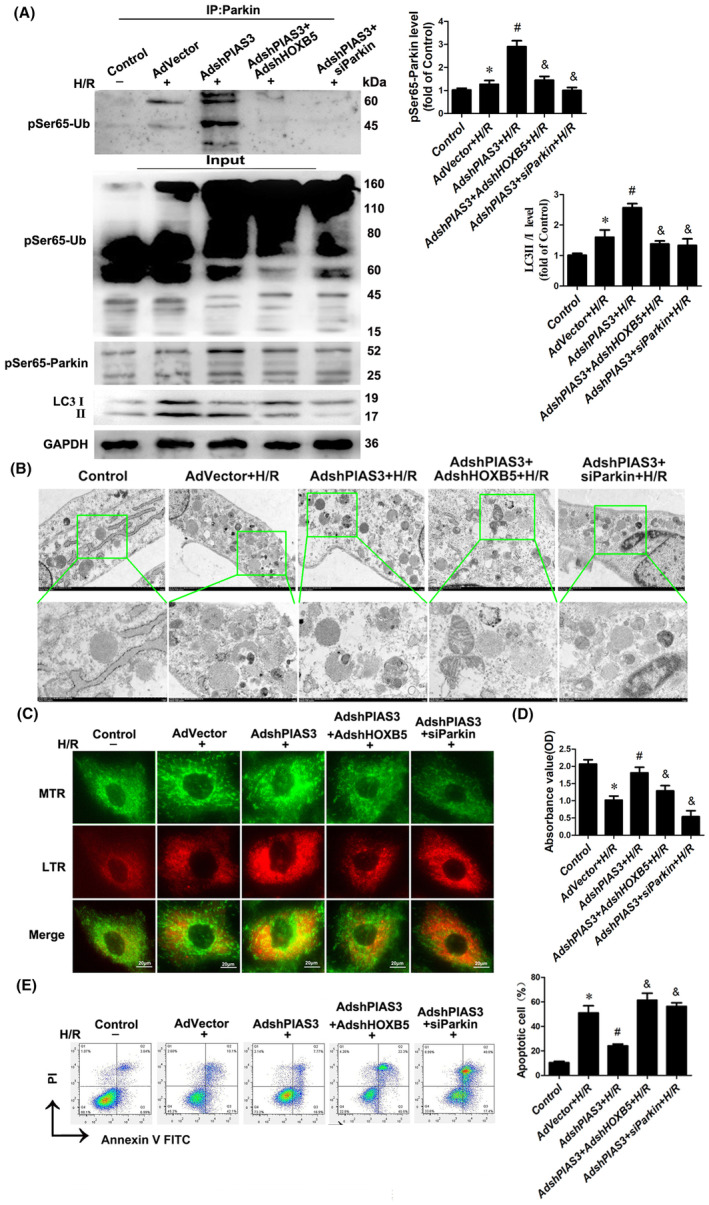
Knockdown of PIAS3 enhances the PINK1/Parkin kinase activity and promotes HOXB5‐mediated mitophagy. (A–E) H9c2 cells were co‐transfected with the adenovirus‐mediated PIAS3 knockdown vector (AdshPIAS3) and the adenovirus‐mediated HOXB5 knockdown vector (AdshHOXB5), or the siRNA‐Parkin (siParkin) for 48 h prior to exposure to H/R. The expression levels of pSer65‐Ub and pSer65‐Parkin, and the LC3‐II/I ratio were examined by Western blotting (A). *n* = 6. *p < 0.05 versus Control, ^#^ *p* < 0.05 versus AdVector+H/R, ^&^ *p* < 0.05 versus AdshPIAS3 + H/R. Electronic micrographs of autophagic vacuoles containing mitochondria (entire or almost degraded) in H9c2 cells (B). Scale bar, 1 μm. Representative images of Mito Tracker (MTR, green fluorescent) and Lyso Tracker (LTR, red fluorescent) stained puncta in H9c2 cells (C). Scale bar, 20 μm. Cell viability was evaluated with the CCK8 Assay Kit (D). *n* = 6. *p < 0.05 versus Control, ^#^ *p* < 0.05 versus AdVector+H/R, ^&^ *p* < 0.05 versus AdshPIAS3 + H/R. Apoptosis rate in H9c2 cells was assessed by flow cytometry (E). *n* = 6. *p < 0.05 versus Control, ^#^ *p* < 0.05 versus AdVector+H/R, ^&^ *p* < 0.05 versus AdshPIAS3 + H/R.

PINK1‐mediated phosphorylation of ubiquitin (pSer65‐Ub) represents the intracellular readouts of PINK1 kinase activity during PINK1‐Parkin‐mediated mitophagy.^15^ As the PIAS3 protein is required to ensure SUMOylation of CARD9 protein and the subsequent binding of CARD9 to HOXB5, we postulated that knockdown of PIAS3 could serve as a positive regulator of mitophagy. Indeed, PINK1 activity was confirmed by an undetectable level of phosphorylated Ub in normal cells, whereas pSer65‐Ub was found to be dramatically enhanced in cells knocked down for PIAS3 after H/R, as measured by immunoblotting (Figure 5A). In contrast, pSer65‐Ub levels were significantly decreased in HOXB5‐ or Parkin‐silenced cells after H/R treatment, and were reduced to the lowest level in Parkin‐deficient cells (Figure 5A). Similar results were obtained for the phosphorylation of Parkin (pSer65‐Parkin) and the LC3II/I ratio in these cells following H/R treatment, consistent with past research demonstrating that Parkin E3 ligase activity is regulated by PINK1 activation (Figure 5A). These data demonstrate that endogenous PINK1 kinase and Parkin E3 ligase activities are functional in cardiomyocytes following H/R.

Electron microscopy revealed that PIAS3 knockdown increased the number of mitochondrial‐autophagic vacuoles following H/R injury, whereas HOXB5 or Parkin deficiency resulted in fewer autophagic vacuoles and mitochondrial swelling or division (Figure 5B). In addition, H/R treatment slightly increased the intensity of yellow light, as evidenced by the co‐localization of mitochondria and lysosomes in the cells. PIAS3 knockdown further increased the intensity of yellow light in the cells after H/R, while interference with HOXB5 or Parkin expression remained at the basal level (Figure 5C). Furthermore, H/R treatment inhibited cell viability and increased apoptosis, while PIAS3 knockdown partially restored cell viability and inhibited H/R‐induced apoptosis, and HOXB5 or Parkin knockdown exacerbated cell death (Figure 5D,E).

### 3.6 Knockdown of PIAS3 alleviates I/R injury

**FIGURE 6:**
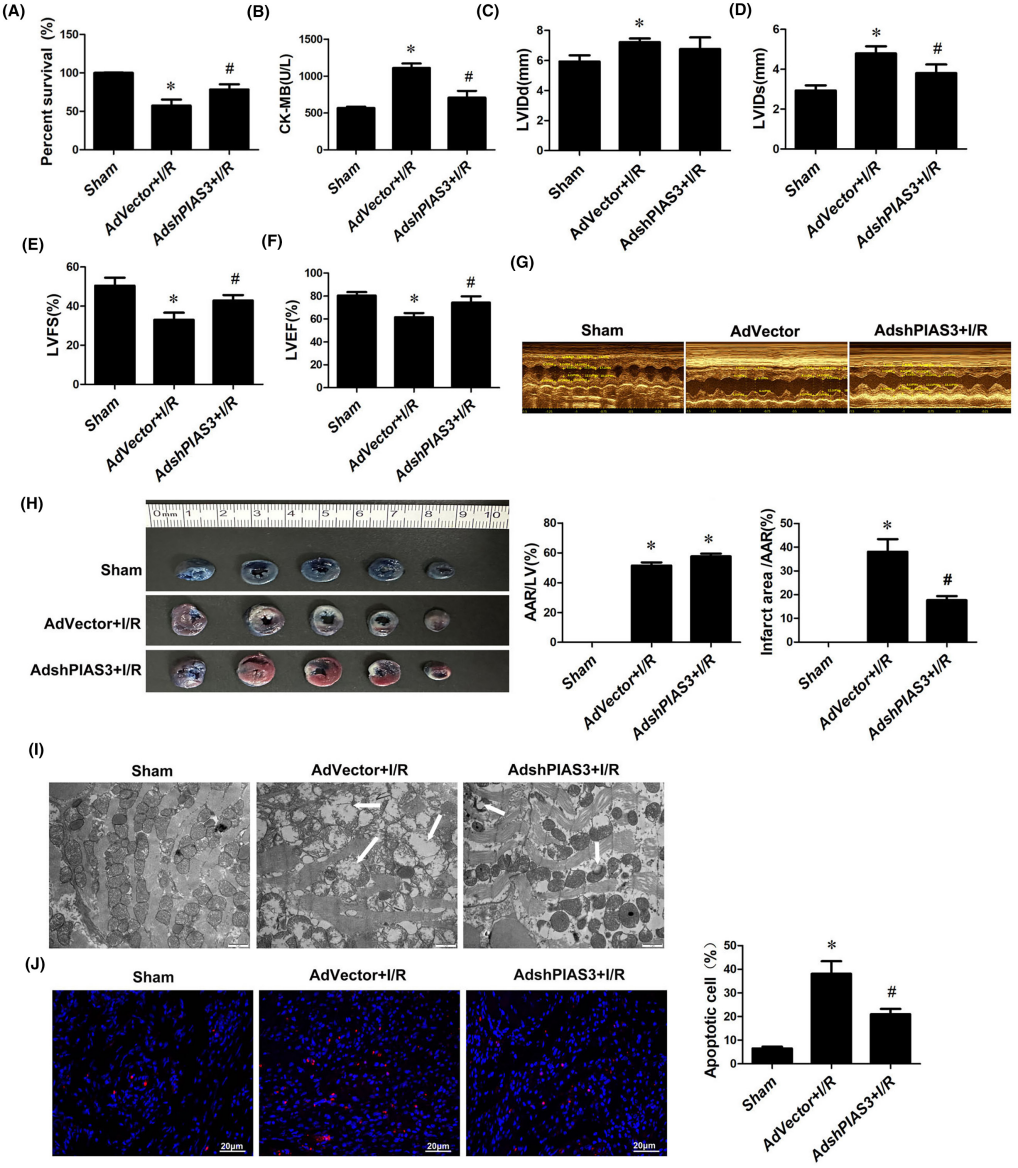
Knockdown of PIAS3 attenuates I/R injury. (A–J) Rat hearts infected with adenovirus‐mediated vector control (AdVector) or adenovirus‐mediated PIAS3 knockdown (AdshPIAS3) vector. Comparison of survival after myocardial I/R in rats (A). *n* = 6. *p < 0.05 versus Sham, ^#^ *p* < 0.05 versus AdVector+I/R. Serum levels of cardiac creatine kinase (CK) at 24 h post‐reperfusion in AdVector and AdshPIAS3 rats (B). *n* = 8. *p < 0.05 versus sham, ^#^ *p* < 0.05 versus AdVector+I/R. Doppler echocardiographic measurement of left ventricular internal dimension diastole (LVIDd) (C), left ventricular internal dimension systole (LVIDs) (D), fractional shortening (E), and ejection fraction (F) in hearts infected with AdVector or AdshHOXB5. *n* = 6. *p < 0.05, *p < 0.05 versus sham, ^#^ *p* < 0.05 versus. AdVector+I/R. Representative images of 2,3,5‐triphenyltetrazolium chloride (TTC)/Evans blue staining and the mean infarct size in I/R (30 min/24 h) hearts in AdVector and AdshPIAS3 infected rats (H). *n* = 6. *p < 0.05 versus sham, ^#^ *p* < 0.05 versus AdVector+I/R. Electron micrographs of autophagic vacuoles containing mitochondria (whole or almost degraded) in AdVector and AdshPIAS3 rat heart tissue subjected to M‐I/R injury (I). Scale bar, 1 μm. Apoptosis in H9c2 cells infected with AdVector and AdshPIAS3 was assessed by TUNEL (J). Scale bar, 20 μm. Apoptotic cells (red) were quantified and analysed. *n* = 6. *p < 0.05 versus sham, ^#^ *p* < 0.05 versus AdVector+I/R.

To determine the biological function of PIAS3 in cardiac function during myocardial I/R injury, rat hearts were infected with an adenovirus‐mediated PIAS3 knockdown (AdshPIAS3) or an adenovirus‐mediated vector control (AdVector). In contrast to the dramatic reduction in survival observed in I/R rats, PIAS3 knockdown improved survival compared to that in the I/R group (Figure 6A). Moreover, serum CK‐MB levels were found to be significantly decreased in PIAS3‐knockdown rats subjected to I/R injury compared to I/R‐injured rats (Figure 6B). Furthermore, we found no difference in LVIDd between I/R and sham rats on echocardiographic examination (Figure 6C). PIAS3 knockdown rats showed significantly improved cardiac function after 24 h of reperfusion, as assessed by LVIDs, LVFS and LVEF, compared to I/R rats (Figure 6D–G). Infarct size was found to be significantly reduced in PIAS3 knockdown rats subjected to I/R injury compared to that in I/R‐injured rats (Figure 6H). Ultrastructurally, the attenuation of myolysis, mitochondrial swelling, disordered arrangement and promotion of mitophagy were observed in PIAS3 knockdown rats in the area at risk of I/R injury (Figure 6I). In addition, the number of TUNEL‐positive cells was found to be significantly reduced in PIAS3 knockdown rats compared to that in I/R rats (Figure 6J). These results strongly suggest that PIAS3 may fatally exacerbate cardiac dysfunction and myocardial injury in vivo.

## 4 DISCUSSION

Acute myocardial infarction (AMI) is a cardiovascular disease that poses a significant threat to life and health.^16^, ^17^ Reperfusion therapy is an effective method to rapidly restore blood flow to the myocardium and reduce ischemic damage; however, reperfusion itself can cause a range of adverse effects.^18^ Based on previous studies, in the present study, we continued investigating the pathological mechanisms of I/R injury and aimed to identify new therapeutic targets for prevention or inhibition. In the present study, we used immunoprecipitation and mitochondrial and lysosomal fluorescence tracking techniques to investigate the regulation of O‐GlcNAc glycosylation of HOXB5 and mitophagy by the CARD9 protein SUMO to provide a theoretical basis to elucidate the mechanism underlying I/R‐induced myocardial injury.

CARD9 is a member of the CARD family of proteins, which are important intracellular bridging proteins that regulate cellular biological functions through protein–protein interactions. The CARD structure binds to other proteins containing the CARD domain (e.g. ASC and BCL10), and plays an important role in the regulation of cellular immunity.^19^ CARD9 competitively binds to the negative regulator of autophagy, Rubicon and disrupts its inhibitory effect on the signalling of UVRAG‐Beclin1‐PI3KC3 and UVRAG‐Vps16‐Rab7, which promotes autophagy and mitigates the death of cardiomyocytes during the I/R process.^4^ This indicates that CARD9 plays a unique biological role within the CARD family of proteins. Our previous study revealed that CARD9 promoted myocardial autophagy during I/R, thus exerting myocardial protection.^4^ Autophagy not only produces ATP by degrading macromolecules to alleviate the energy crisis caused by mitochondrial dysfunction following ischemia, but also maintains intracellular protein homeostasis by removing damaged proteins.^20^, ^21^ Following I/R, the mitochondria in cardiomyocytes become dysfunctional, the mitochondrial membrane potential decreases, membrane permeability increases, and the permeability of the mitochondrial membrane increases. This leads to the release of cytochrome C (Cyt‐c), which initiates apoptotic signalling mediated by mitochondrial damage. However, mitophagy can remove damaged mitochondria from cells to prevent apoptosis.^2^

To further identify the biological functions of CARD9, we performed immunoprecipitation to extract proteins bound to CARD9 in cardiomyocytes, and further analysed these proteins using protein profiling (data not shown). The results revealed that CARD9 binds to various transcription factors, including HOXB5, HMBOX1, ZNF655 and ZNF774. However, it is noteworthy that transcription factors function in the nucleus, whereas CARD9 proteins are predominantly distributed in the cytoplasm. One plausible explanation for this is that the binding of CARD9 to these transcription factors may prevent their translocation to the nucleus. Indeed, many transcription factors, such as NF‐κB(p65), Gli2, SMAD4, are ‘sequestered’ in the cytosol in an inactivated state.^22^, ^23^, ^24^ Only when upstream signalling pathways, such as NF‐κB, Hedgehog, TGF‐β, are activated, these transcription factors can dissociate from the ‘repressor proteins’ in the cytosol and enter the nucleus to perform their transcriptional functions.^22^, ^23^, ^24^ As such, we hypothesized that CARD9 may also act as a ‘repressor protein’, preventing the entry of transcription factors into the nucleus and thereby inhibiting the transcription and expression of relevant target genes.

Downregulation of HOXB5 expression prevents the transcription of SOX9, leading to neural crest apoptosis and sympathetic and dorsal root ganglion dysplasia.^5^ In addition, HOXB5 activates multiple pro‐proliferative and anti‐apoptotic signals in cells, such as the GSK3β/β‐catenin and ERK/MDM2 signalling pathways, to promote cell survival and growth.^6^, ^7^ In the present study, we found that HOXB5 overexpression promoted mitophagy in cardiomyocytes following H/R treatment (Figure 3). Given that HOXB5 has been proposed as a potential transcription factor that regulates the Parkin gene, which in turn is a key factor in initiating mitophagy, it is possible that HOXB5 promotes mitophagy in cardiomyocytes following H/R by elevating Parkin expression.

O‐GlcNAc glycosylation involves the attachment of GlcNAc from UDP‐GlcNAc to the oxygen atom of the hydroxyl group of serine or threonine via O‐GlcNAc transferase (OGT). UDP‐GlcNAc is the only donor for O‐GlcNAc glycosylation of proteins, and cellular UDP‐GlcNAc is mainly synthesized from glucose through the hexosamine biosynthesis pathway. Interestingly, I/R promoted the modification of protein O‐GlcNAc glycosylation, rather than inhibiting it, mainly because only 0.003%–0.006% of cellular glucose was used to synthesize UDP‐GlcNAc; as such, reducing cellular glucose had little effect on UDP‐GlcNAc synthesis. Furthermore, I/R induces OGT expression, which promotes O‐GlcNAc glycosylation.^11^, ^25^ A growing number of studies have further confirmed that the inhibition of O‐GlcNAc glycosylation exacerbates I/R‐induced myocardial injury; conversely, the induction of O‐GlcNAc glycosylation alleviates I/R‐induced myocardial injury.^10^, ^25^ O‐GlcNAc glycosylation has also been confirmed to be an important signal that induces proteoplasmic‐nuclear transport.^18^ O‐GlcNAc glycosylation modification induces the translocation of hnRNP‐K from the cytoplasm to the nucleus, and has also been shown to affect the translocation of NF‐κB to the nucleus.^26^, ^27^ O‐GlcNAc glycosylation primarily induces the nuclear translocation of proteins, which may be associated with the recognition of nuclear plasma transport proteins and regulation of nuclear pore complexes.^9^, ^28^ Bioinformatic analysis has previously suggested that HOXB5 contains multiple O‐GlcNAc glycosylation modification sites, but the O‐GlcNAc glycosylation modification site is partially occupied by the binding region of CARD9 to HOXB5 (data not shown). Analyses revealed that CARD9 binds to HOXB5 and prevents it from translocating from the cytoplasm to the nucleus to induce Parkin expression and mitophagy following I/R. However, we previously reported that CARD9 everts a cardioprotective effect, and that it is inappropriate to activate the function of HOXB5 by knocking down CARD9. Although the overall level of O‐GlcNAc glycosylation in cardiomyocytes increased after I/R, several O‐GlcNAc glycosylation modification sites on HOXB5 may be occupied by CARD9, thus interfering with HOXB5 glycosylation and inhibiting HOXB5 translocation to the nucleus. In the present study, we used the unique approach of knocking down PIAS3 to downregulate the SUMO level of the CARD9 protein to promote HOXB5‐induced mitophagy and alleviate I/R‐induced myocardial injury.

SUMOylation is defined as the process by which an E3 ligase transfers a SUMO molecule from the E2 conjugating enzyme to the lysine of a target protein. The SUMO molecule on the target protein binds to the SUMO‐interactive motif (SIM) of other proteins, thus facilitating the binding of SUMO‐modified proteins to other proteins.^8^, ^12^ Bioinformatics analysis further revealed that the CARD9 protein contains multiple SUMO modification sites, whereas the HOXB5 protein contains SIM (data not shown). Thus, SUMO on CARD9 may facilitate binding between CARD9 and HOXB5 by binding to HOXB5 on SIM. Numerous studies have further reported elevated SUMO levels in many proteins following myocardial I/R.^29^, ^30^ Consistent with these findings, we found that the SUMO levels of CARD9 were upregulated in cardiomyocytes following I/R (Figure 1). It has also been reported that PIAS3 reduces the activated form of STAT3, increases the activity of the downstream Nestin/Nrf2/HO‐1 pathway, and inhibits damage in Alzheimer's disease model SH‐SY5Y cells.^31^ Recently, increased PIAS3 expression levels were found to reduce LV systolic percentage and ejection fraction values and increase infarct size in a rat model of I/R,^32^ indicating that PIAS3 may be a novel target for the inhibition of myocardial I/R injury. Knockdown of PIAS3 in vivo has further been shown to downregulate the SUMO levels of CARD9 after myocardial I/R and to further decrease the levels of HOXB5 on CARD9, resulting in increased O‐GlcNAc glycosylation (Figure 1). Interestingly, we found that knockdown of PIAS3 enhances PINK1/Parkin kinase activity and promoted mitophagy (Figure 5). This evidence indicates that SUMO modification of the CARD9 protein promotes CARD9 binding to HOXB5, but inhibits HOXB5 O‐GlcNAc glycosylation, disturbing PINK1/Parkin signalling‐induced mitophagy.

**FIGURE 7:**
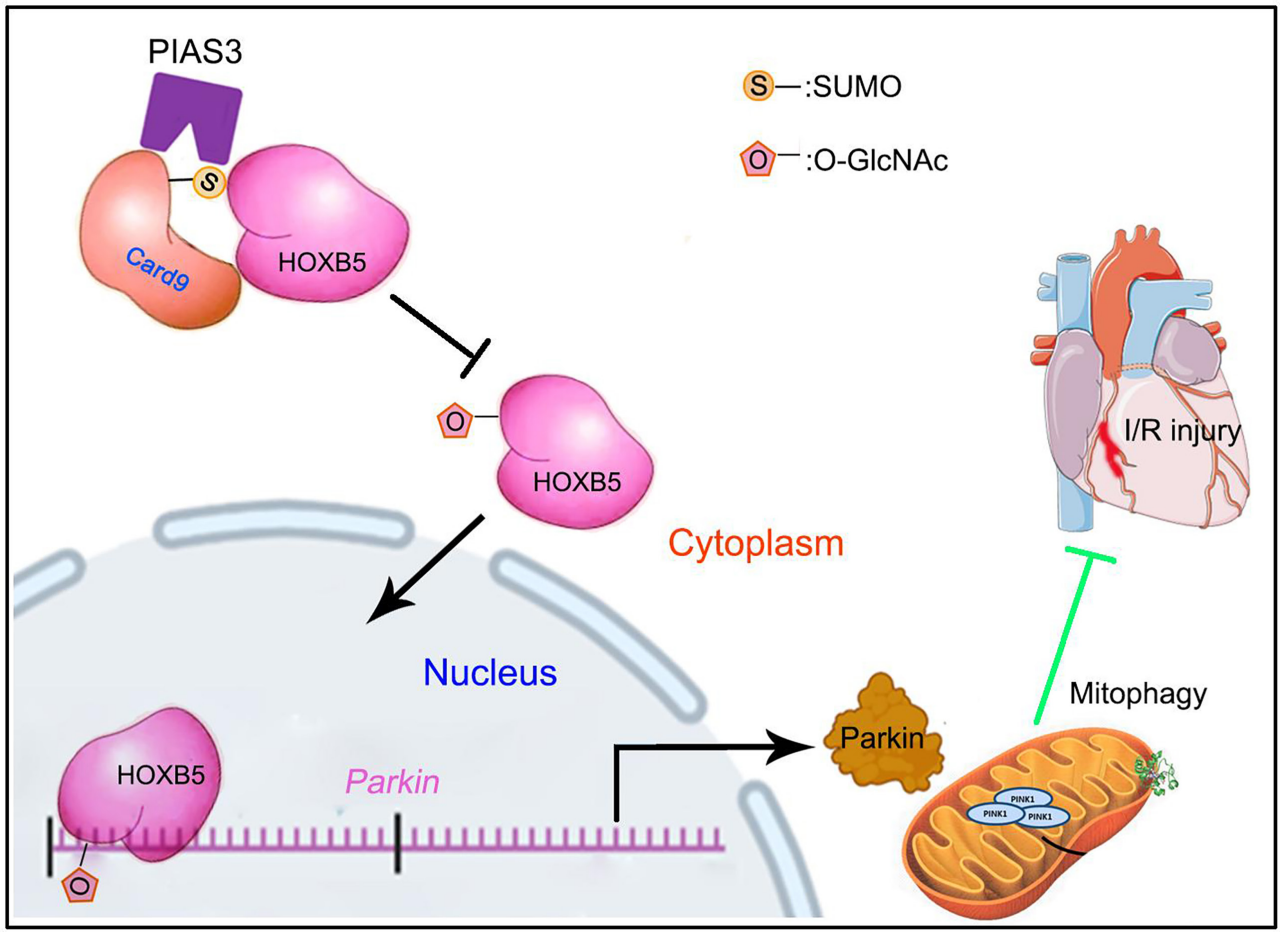
Diagram illustrating the simplified mechanism at the SUMO level of the role of the CARD9 protein in the formation of mitophagy. SUMOylation regulates the binding of CARD9 to HOXB5, which in turn affects the O‐GlcNAc glycosylation of HOXB5 proteins and the nuclear translocation of HOXB5, thereby regulating Parkin‐mediated mitophagy and attenuating I/R injury.

In conclusion, these data demonstrate that SUMO modification regulates the binding of CARD9 to HOXB5, which in turn affects O‐GlcNAc glycosylation of HOXB5 proteins. Further, we found that knockdown PIAS3 can facilitate the nuclear translocation of HOXB5 and promote Parkin‐mediated mitophagy to alleviate I/R‐induced myocardial injury. The diagram illustrating the mechanism for the role of the CARD9‐SUMO‐modification in the formation of mitophagy in I/R‐induced myocardial injury (Figure 7).

## AUTHOR CONTRIBUTIONS

**Yuanbin Li:** Conceptualization (equal); data curation (equal); funding acquisition (equal); methodology (equal); resources (equal); validation (equal); visualization (equal); writing – original draft (equal); writing – review and editing (equal). **Yuting Tang:** Data curation (equal); formal analysis (equal); investigation (equal); methodology (equal); project administration (equal); software (equal); supervision (equal). **Xu Yan:** Formal analysis (equal); investigation (equal); methodology (equal); project administration (equal); resources (equal); software (equal); supervision (equal). **Hui Lin:** Conceptualization (equal); data curation (equal); investigation (equal); resources (equal); software (equal). **Wanjin Jiang:** Investigation (equal); methodology (equal); project administration (equal); resources (equal); software (equal). **Hu Zhao:** Formal analysis (equal); investigation (equal); methodology (equal); project administration (equal). **Luwei Zhang:** Conceptualization (equal); methodology (equal); project administration (equal); validation (equal); visualization (equal); writing – review and editing (equal). **Zhuang Chen:** Conceptualization (equal); funding acquisition (equal); resources (equal); validation (equal); writing – original draft (equal); writing – review and editing (equal).

## CONFLICT OF INTEREST STATEMENT

The authors declare that they have no conficts of interest with the contents of this article.

## Data Availability

The data that support the findings of this study are available from the corresponding author upon reasonable request.
